# Case Report: Primary giant chondrosarcoma of the manubrium sterni

**DOI:** 10.3389/fsurg.2026.1779653

**Published:** 2026-04-29

**Authors:** Yinghao Zhu, Yang Lou, Xianguo Chen

**Affiliations:** Department of Cardiothoracic Surgery, Affiliated Jinhua Hospital, Zhejiang University School of Medicine, Jinhua, Zhejiang, China

**Keywords:** bone malignant tumor, case report, chest wall reconstruction, chondrosarcoma, manubrium sterni

## Abstract

Chondrosarcoma is one of the most common primary malignant bone tumors in adults, with a predilection for sites such as the pelvis and femur. This article reports a rare case of giant chondrosarcoma originating from the manubrium sterni in a 42-year-old male patient. The patient presented to the outpatient clinic with chest pain accompanied by chest wall swelling. Physical examination revealed a firm mass at the manubrium, and chest CT demonstrated osteolytic destruction of the sternum. Needle biopsy pathology was suggestive of chondrosarcoma. A multidisciplinary team performed a radical resection of the sternal tumor. The postoperative outcome was favorable, with a stable thoracic cage. Histopathological examination confirmed the diagnosis of grade II chondrosarcoma.

## Introduction

Chondrosarcoma is an uncommon mesenchymal malignancy, accounting for 20%–40% of all primary bone tumors, with an estimated annual incidence of 0.5 per 100,000 population (1). In contrast to osteosarcoma or Ewing sarcoma, which predominate in adolescents and young adults, the incidence of chondrosarcoma increases with advancing age, peaking between the fourth and sixth decades (2). With a male-to-female ratio of approximately 1.5–2:1. Primary chondrosarcoma of the sternum is exceptionally rare, representing <0.2% (3).

Computed tomography (CT) is the primary screening modality for chondrosarcoma. On CT, the tumor typically manifests as osteolytic bone destruction, chondroid matrix calcification, osseous expansion with cortical alterations, and an associated soft-tissue mass (4). Magnetic resonance imaging (MRI) is essential in the diagnosis and management of chondrosarcoma. Its high soft-tissue contrast and multiplanar capability enable accurate assessment of intramedullary extent, cortical destruction, extraosseous extension, and neurovascular involvement (5). T2-weighted hyperintensity—reflecting cartilaginous matrix—and enhancement patterns help distinguish malignant from benign cartilaginous tumors (6). Combined with CT—which better visualizes calcifications and bone integrity—MRI forms the foundation of multimodal imaging for chondrosarcoma. Nevertheless, histopathological confirmation is still required before surgical intervention. The histological demonstration of lobular cartilaginous tissue with intramedullary infiltration and cellular atypia remains the gold standard for diagnosis (7).

Given the potential of chondrosarcoma for progressive local destruction and distant metastasis, and the limited efficacy of conventional chemotherapy and radiotherapy for most subtypes, timely surgical intervention is crucial following diagnosis (8). The specific surgical approach and extent of resection are primarily determined by the tumor's anatomical location and its histological grade (9).

## Case report

**Figure 1:**
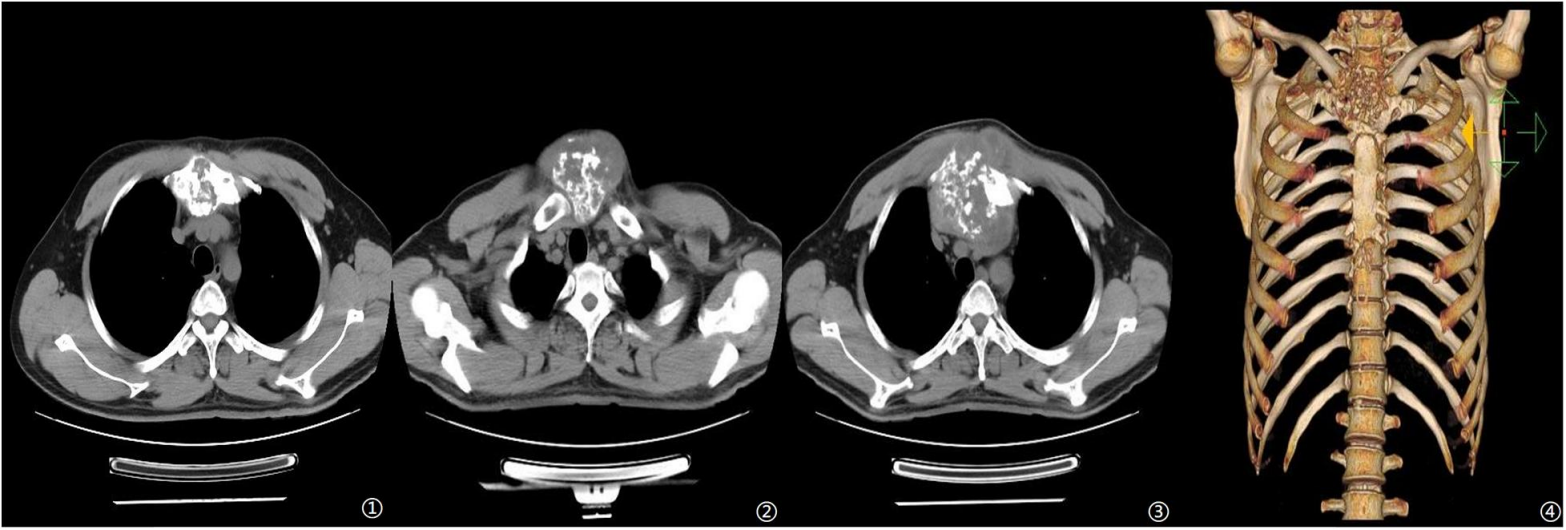
CT before operation. ① Initial CT scan in 2023. ②③④ Preoperative CT scan and Three-dimensional reconstruction CT.

A 42-year-old man presented to the outpatient clinic with anterior chest-wall swelling and chest pain. Review of his medical history revealed that a chest CT performed in August 2023 had already indicated bone destruction of the manubrium sterni, (Figure 1); however, the patient declined treatment at that time. For the following two years, the patient underwent no further examinations or medical consultations. In November 2025, the patient returned to our hospital outpatient department due to progression of the anterior chest wall swelling and chest pain, which had become intolerable, and was subsequently admitted.

Physical examination revealed a palpable mass measuring approximately 9 × 9 cm at the manubrium sterni, with no detectable pulsation. Admission laboratory studies, including a comprehensive panel of serum tumor markers, were within normal limits. Contrast-enhanced CT demonstrated bone destruction of the manubrium sterni accompanied by the formation of a soft tissue mass, with the lesion involving the clavicle, the sternal body, and the first and second ribs. SPECT revealed increased metabolic activity within the manubrium sterni.

We established a multidisciplinary team (MDT) comprising specialists from thoracic surgery, orthopedics, pathology, radiology, medical oncology, and radiation oncology. Regarding the choice of biopsy approach, the patient presents with prominent imaging features suggestive of chondrosarcoma. Given that open biopsy is associated with greater invasiveness and a higher risk of complications compared with percutaneous needle biopsy, we decided to perform image-guided percutaneous needle biopsy as the initial procedure. Open biopsy will be reserved only if a definitive histopathological diagnosis cannot be achieved with percutaneous needle biopsy. A needle biopsy was subsequently performed. Microscopic examination revealed a myxoid chondroid matrix and atypical cells, confirming the diagnosis of chondrosarcoma. Consequently, a radical sternectomy was performed.

During the operation, the tumor was observed to have invaded the clavicles, the sternal body, and the first and second ribs. We resected the medial one-third of both clavicles, the superior one-third of the sternal body, along with the anterior portions of the first and second ribs and the involved musculature. Fortunately, the tumor did not involve the aortic arch or the innominate vein, thus eliminating the need for cardiopulmonary bypass or prosthetic vascular graft replacement. No significant intraoperative hemorrhage occurred.

**Figure 2:**
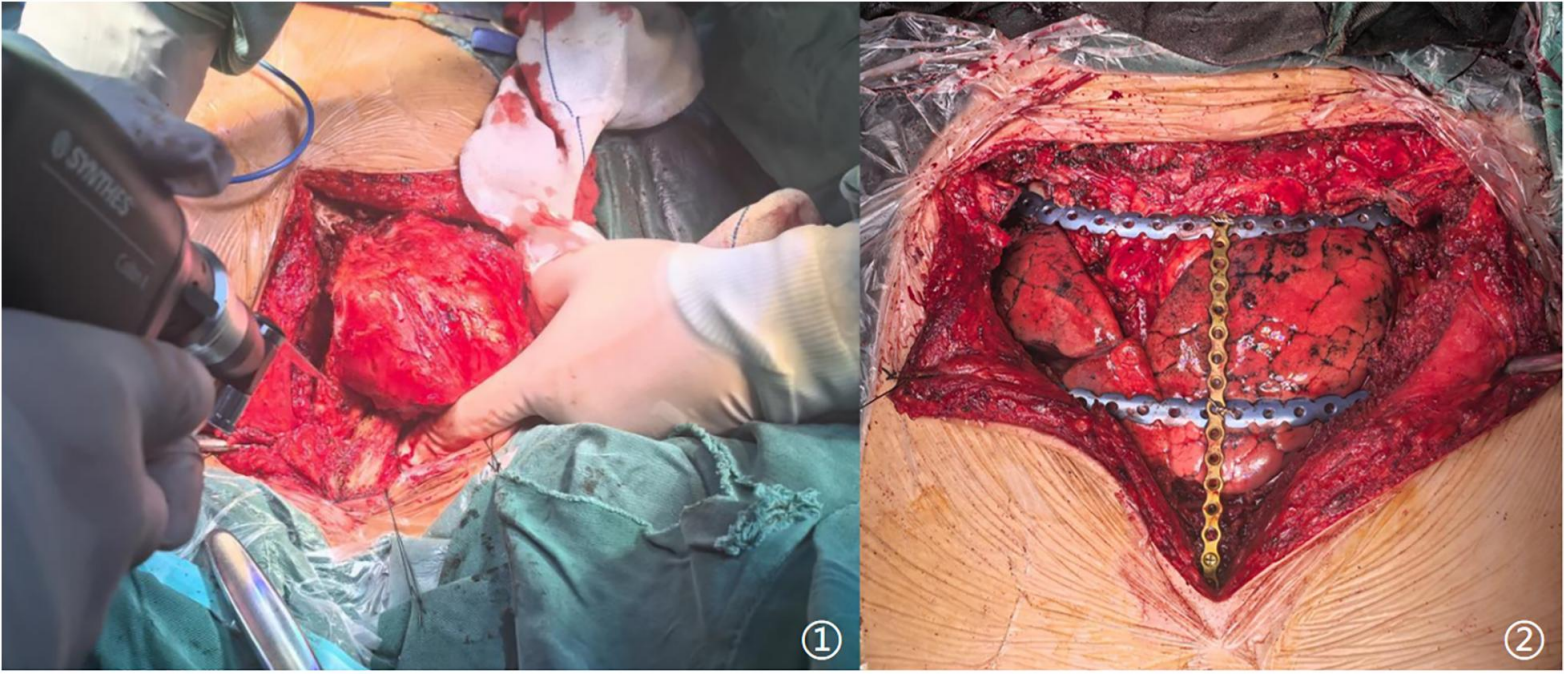
Intraoperative aspect. ① Radical resection of Chondrosarcoma ② chest wall reconstruction by three titanium plates.

**Figure 3:**
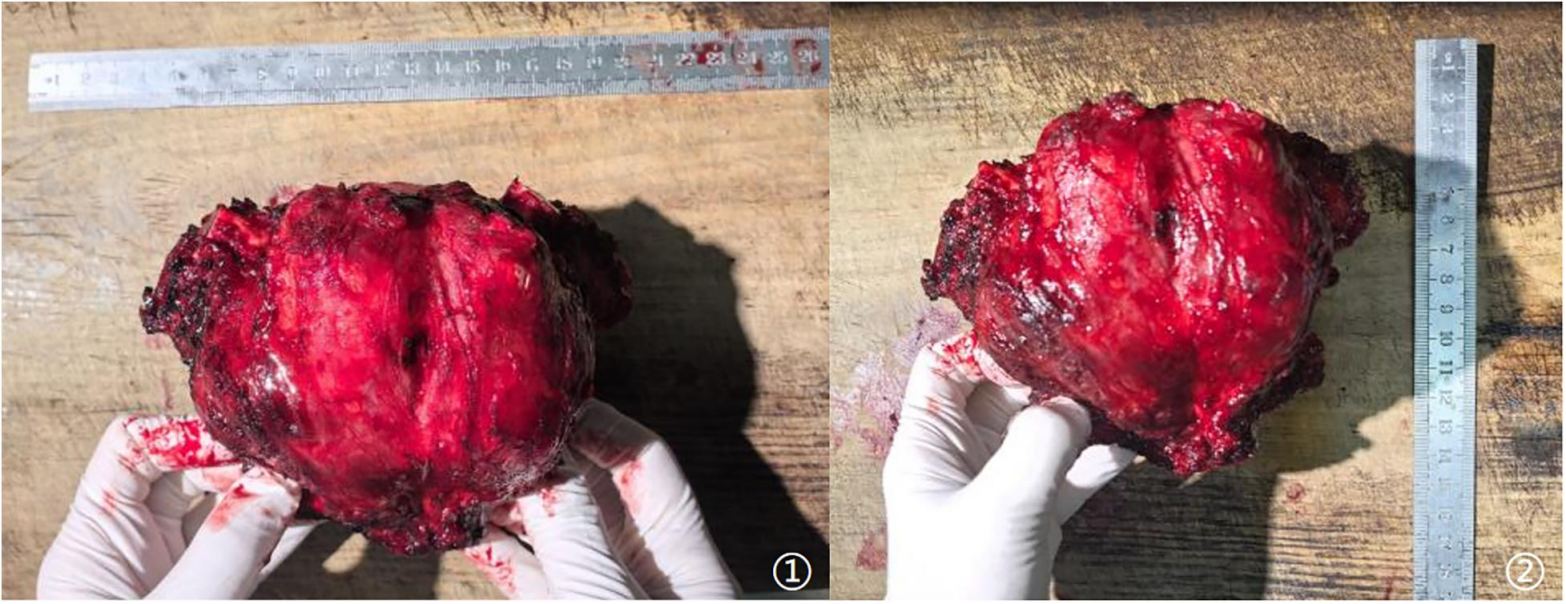
Tumor size ① the length of the specimen ② the width of the specimen.

Chest-wall stability was restored with a single sternal titanium plate and two rib plates secured to the residual sternal and rib stumps (Figure 2). Since the tumor did not extensively involve the muscular tissue, the chest incision could be closed directly. The tumor size is about 15*12 cm (Figure 3).

**Figure 4:**
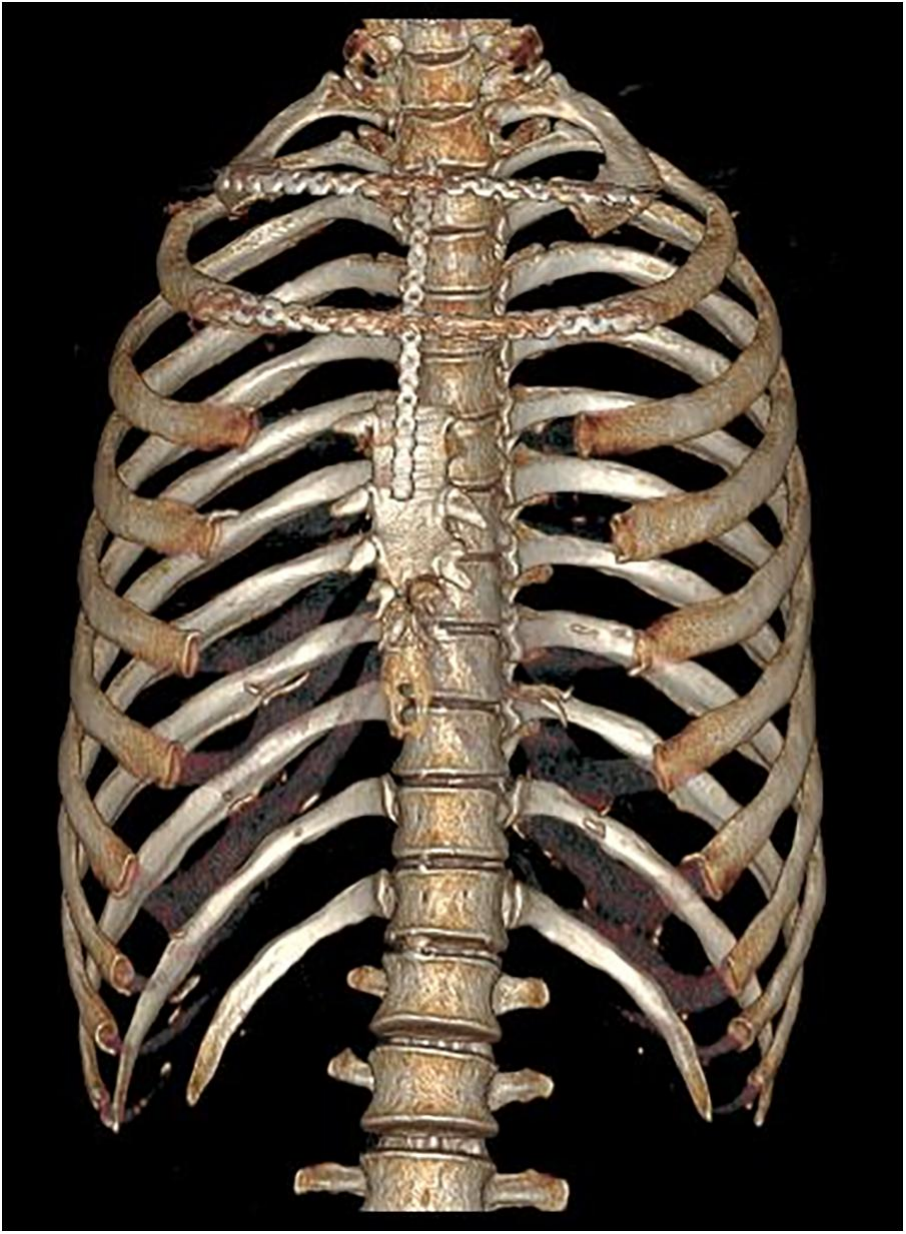
Postoperative three-dimensional reconstruction CT.

Postoperatively the patient maintained stable thorax without paradoxical respiration. CT with Three-dimensional rib reconstruction confirmed correct plate positioning (Figure 4). Definitive histopathology established a grade II chondrosarcoma with clear resection margins.

One month after surgery, outpatient follow-up CT imaging demonstrated that the titanium plate was in place with no evidence of significant tumor recurrence. The patient was advised to the Department of Radiation Oncology for adjuvant local radiotherapy.

## Discussion

Chondrosarcoma is a rare malignant tumor originating from the chondroid differentiation lineage (10, 11). Although it accounts for only 15%–25% of all primary bone tumors, it ranks as the second most common malignant bone tumor in adults (12). It is a malignant hyaline cartilage tumor characterized by diverse morphological features and clinical behaviors. The overall five-year disease-specific survival rate is approximately 70%; histological grade is the most powerful predictor of survival, 90%, 81%, and 29% for grades 1, 2, and 3 chondrosarcoma, respectively. Once distant metastasis occurs, the associated mortality rate rises sharply to 70%–100%, with a median survival of less than one year (13).

It is now recognized that chondrosarcoma does not arise from a single pathogenic pathway but rather results from the interaction of multiple genetic and microenvironmental factors. Approximately 10%–15% of cases are associated with Ollier disease or Maffucci syndrome, both of which harbor somatic *IDH1/2* mutations and carry a lifetime cumulative risk of 15%–35% (12). In sporadic chondrosarcomas, a triad of key genetic drivers has been identified: IDH1-R132H, TERT promoter, and COL2A1 mutations. Among these, IDH mutations lead to the accumulation of 2-hydroxyglutarate (2-HG), resulting in epigenetic dysregulation that suppresses chondrocyte differentiation (14). The inherently avascular nature of the cartilage matrix creates a hypoxic and lactate-rich microenvironment, which further induces the expression of immune checkpoint molecules such as PD-L1 and CD47. Notably, cumulative CT radiation exposure during childhood (≥50 mGy) is associated with a 2–3 fold increased risk of developing chondrosarcoma, while no causal link has been established with prior trauma or internal fixation (15).

Early-stage Chondrosarcoma lesions may present with only vague symptoms such as dull pain or “nocturnal aching”. In contrast, high-grade or dedifferentiated subtypes manifest as progressive severe pain, with the Visual Analogue Scale (VAS) score potentially escalating to ≥4 within 1–3 months (16). Deep-seated lesions in the pelvis or shoulder girdle often remain occult until exceeding 5 cm in size, whereas masses in the distal femur tend to present earlier as “rock-hard, well-defined” lumps. The imaging triad consists of (i) radiographic “rings-and-arcs” calcification coupled with endosteal scalloping, (ii) CT demonstration of intramedullary “popcorn” calcification, and (iii) MRI revealing extremely high T2 signal (cartilage water content >80%), where the presence of “low-signal septations” suggests a high-grade lesion (17). The radiographic features of low-grade chondrosarcoma are often very similar to those of enchondroma (18). Biopsy is warranted if any one of the following four criteria is met: lesion size >6 cm, presence of pain, cortical involvement >2/3 of the thickness, or periosteal reaction. In immunohistochemistry, the IDH1-R132H antibody can yield results within 20 min, a positive result is sufficient to upgrade a diagnosis from “enchondroma” to “chondrosarcoma (IDH-mutant type)" (19). Clinical management therefore necessitates an integrated decision-making approach based on the clinico-radiologic-pathologic triad.

For the histopathological confirmation of chest wall tumors, open biopsy remains the gold standard (20). It enables direct visualization and acquisition of ample, representative tissue samples, providing high diagnostic accuracy—particularly in lesions exhibiting marked histological heterogeneity or atypical imaging features. However, it carries greater procedural risks, including infection, nerve injury, and tumor seeding along the biopsy tract (21). Furthermore, if the biopsy incision is not aligned with the planned surgical approach for definitive resection, it may compromise subsequent oncologic surgery (22). In contrast, percutaneous needle biopsy offers distinct advantages, including minimal invasiveness, rapid recovery, fewer complications, and the ability to be performed under local anesthesia (23). With contemporary techniques, it achieves high diagnostic accuracy for most bone tumors. Nevertheless, its limited tissue yield increases the risk of sampling error, often necessitating repeat procedures and potentially delaying definitive diagnosis (24). Thus, both open and percutaneous biopsy approaches have distinct strengths and limitations in the evaluation of chest wall tumors. Based on current evidence, percutaneous needle biopsy—especially when performed under image guidance—is generally recommended as the initial diagnostic modality. Open biopsy should be reserved for cases in which percutaneous biopsy yields inconclusive results or when clinical and imaging findings raise high suspicion of malignancy despite negative or ambiguous histology (25).

Chondrosarcoma is histologically graded I–III based on cellularity, nuclear atypia, mitotic activity, and matrix features: Grade I is low-grade with mild atypia and abundant cartilage matrix; Grade II shows moderate atypia and cellularity; Grade III exhibits marked pleomorphism, high cellularity, frequent mitoses, and necrosis (26). Dedifferentiated chondrosarcoma contains a high-grade non-cartilaginous sarcomatous component. Surgical margins are classified as intralesional, marginal, wide, or radical; wide or radical resection (with ≥1 cm of normal tissue) is preferred for Grade II–III tumors to reduce recurrence risk. Margin status is confirmed histologically on permanent sections, as frozen sections are unreliable in cartilaginous tissue (27, 28).

The Enneking surgical staging system remains the gold standard. radical resection can reduce the local recurrence rate from 40% to <10% (29, 30). Radical resection involves extensive surgical margins and necessitates meticulous protection of surrounding tissues (31). Taking the case of manubrial chondrosarcoma described in this article as an example, bilateral clavicular involvement mandates careful preservation of the brachial plexus; otherwise, upper limb mobility may be significantly compromised. Additionally, critical vascular structures posterior to the sternum must be safeguarded to prevent catastrophic hemorrhage. Following resection of the sternum and involved ribs, thoracic wall reconstruction is essential to maintain structural integrity and ensure normal postoperative respiratory function.

Chest wall reconstruction requires a multidisciplinary, individualized approach based on the size, location, depth (bony vs. soft tissue), etiology (e.g., tumor resection, trauma, infection), and patient factors (32). The primary goals are to restore structural stability, prevent paradoxical respiration, obliterate dead space, and provide durable soft-tissue coverage (33). Bony defects larger than 5–6 cm or involving ≥2 ribs typically necessitate rigid prosthetic support using materials such as Gore-Tex, polypropylene mesh, or titanium plates (33). This is followed by well-vascularized soft-tissue coverage—commonly with pedicled flaps like the latissimus dorsi, pectoralis major, rectus abdominis, or omentum—to reduce infection risk and ensure integration of the implant (34). In select cases, microvascular free flaps (e.g., DIEP) may be used for combined functional and aesthetic reconstruction, particularly after oncologic resection (35). Postoperative management focuses on chest drainage, respiratory support, and flap monitoring to optimize outcomes (36).

Chondrosarcomas are generally resistant to radiotherapy and chemotherapy (37). Stereotactic body radiotherapy (SBRT) is employed only for unresectable skull base or spinal cases, with a regimen of 70 Gy in 10 fractions achieving a 3-year local control rate of 72%; however, 12% of cases develop radiation myelitis (38). Regarding targeted therapy, the IDH1 inhibitor ivosidenib demonstrates an objective response rate of 12% and a disease control rate of 56% (39). When combined with a PD-1 inhibitor, peripheral blood T-cell receptor (TCR) diversity increased by 2.7-fold, and a Phase III clinical trial is currently underway (40, 41).

Postoperative sternal wound infection requires close monitoring, particularly due to the presence of titanium plate implants. Chronic sternal osteomyelitis or implant-related infection can be a cause of postoperative mortality (42). The use of antibiotics and adequate drainage are crucial therapeutic measures. Additionally, dislodgement of fixation screws may lead to chest wall instability and paradoxical breathing, potentially resulting in decreased SpO₂. The onset of chest wall instability or paradoxical breathing necessitates immediate bedside x-ray examination, with secondary surgical fixation performed if required. Atelectasis and pulmonary infection are also potential postoperative complications. Therefore, regular wound dressing changes and periodic x-ray reviews are essential for monitoring wound healing, as well as pulmonary and thoracic conditions, enabling timely intervention should any related complications arise.

In this case, the patient with chondrosarcoma of the manubrium sterni, although the tumor involved the clavicle, first rib, and second rib, fortunately did not invade any major blood vessels. The patient underwent radical resection followed by thoracic reconstruction using titanium plates. Currently, the postoperative incision is healing well, and the thoracic cage remains stable.

## Conclusion

This article reports a giant chondrosarcoma of the manubrium sterni, which invaded the bilateral clavicles, the body of the sternum, and the bilateral first and second ribs. A favourable outcome was achieved through comprehensive pre-operative evaluation, radical resection with reconstruction of a stable thoracic cage, and meticulous post-operative nursing surveillance.

## Data Availability

The raw data supporting the conclusions of this article will be made available by the authors, without undue reservation.
